# Immunosuppressive effects of *Amblyomma cajennense* tick saliva on murine bone marrow-derived dendritic cells

**DOI:** 10.1186/s13071-015-0634-7

**Published:** 2015-01-14

**Authors:** Tamires Marielem Carvalho-Costa, Maria Tays Mendes, Marcos Vinicius da Silva, Thiago Alvares da Costa, Monique Gomes Salles Tiburcio, Ana Carolina Borella Marfil Anhê, Virmondes Rodrigues, Carlo Jose Freire Oliveira

**Affiliations:** Graduate Course of Physiological Sciences, Laboratory of Immunology, Federal University of Triângulo Mineiro, Uberaba, Minas Gerais Brazil; Graduate Course of Tropical Medicine and Infectology, Laboratory of Immunology, Federal University of Triângulo Mineiro, Av. Getúlio Guaritá S/N, Uberaba, Minas Gerais 38015-050 Brazil; Undergraduate Course of Environmental Engineering, Federal University of Triângulo Mineiro, Uberaba, Minas Gerais Brazil

**Keywords:** Ticks, *Amblyomma cajennense*, Saliva, Dendritic cells

## Abstract

**Background:**

Dendritic cells (DCs) are professional antigen-presenting cells with vital roles in the activation of host immunity. Ticks are bloodsucking arthropods that secrete bioactive compounds with immunomodulatory properties via their saliva. It is known that some tick species modulate the biology of DCs with different intensities; however, studies on *Amblyomma cajennense*, the Cayenne tick, have not yet been performed, although this species is considered one of the most capable of modulating immune responses of different hosts.

**Methods:**

Engorged female ticks were stimulated with dopamine to induce salivation, and saliva was pooled. The effects of tick saliva on the biology of dendritic cells were assessed by examining DC differentiation, maturation, migration, cellular viability, cytokine production and expression of surface markers by flow cytometry and ELISA. Competitive enzyme immunoassays (EIA) were used to measure saliva prostaglandin-E_2_ (PGE_2_). Statistical significance was determined by ANOVA followed by Tukey’s post-test or by the Kruskal-Wallis test with the Dunns post-test.

**Results:**

In this work, we demonstrated that the presence of *A. cajennense* saliva to bone marrow cultures inhibit DC differentiation. This inhibition was not accompanied by inhibition or induction of stimulatory and co-stimulatory molecules such as MHC-II, CD40, CD80 or CD86. Immature and mature DCs that were pre-exposed to saliva showed reduced migration toward the chemokines RANTES and MIP-3β. This inhibition was associated to a reduced expression of CCR5 (the receptor for RANTES) or CCR7 (the receptor for MIP-3β) induced by the presence of saliva in the cultures. Tick saliva also inhibited IL-12p40, IL-6 and TNF-α in a concentration-dependent manner while potentiating IL-10 cytokine production by DCs stimulated with Toll-like receptor-4 ligand. Additionally*, A. cajennense* tick saliva inhibited the expression of CD40 and CD86 in mature DCs while potentiating the expression of PD-L1. PGE_2_ was detected as one of the constituents of saliva at a concentration of ~ 80 ng/ml, and we believe that most of the results reported herein are due to the presence of PGE_2_.

**Conclusions:**

These results help to understand the tick-host interaction and demonstrate that *A. cajennense* ticks appear to have mechanisms for modulating host immune cells, including DCs.

## Background

The secretion of biologically active substances through saliva is one of the most important evolutionary characteristics of ticks; it facilitates an effective interaction with the host, allowing ticks to feed and favoring transmission of the pathogens that they carry [1,2]. Among the molecules present in tick saliva, those that influence hemostasis, inflammation, and host immunity are considered the most important in modulating the tick-host interaction [2-5]. The substances continuously released by ticks during blood feeding may be different depending on the species and the stage of the tick [4,6], and these differences among the species have proven to be vital to the understanding of host preference, feeding time, capacity for modulating hemostatic and immune barriers, and diversity of pathogens transmitted by each tick species.

The *Amblyomma cajennense* tick, or the Cayenne tick, is the main vector of equine babesiosis and human spotted fever in Brazil. It is considered one of the species with the greatest potential to subvert the defenses of their hosts. Despite having horses as their main hosts, these ticks can sustain themselves in areas free of equine; especially the larval and nymphal stages, which are able to parasitize any domestic or wild mammal, birds and even humans [7-10]. As the immune responses of their different hosts – including that of humans – vary considerably, it is reasonable to assume that the molecular composition of the saliva of this tick species is sufficiently complex to modulate each host’s immune response.

Given the above assumption, the molecular composition of the saliva of *A. cajennense* has aroused the interest of many scientists [11], and studies involving this tick species and host immune responses have been published in recent years [12]. Dogs, horses and woolless lambs infested by *A. cajennense* ticks only acquire partial resistance even after repeated infestations because only some biological parameters of these ticks are negatively affected by immune response [13-16]. Similarly, experimental results showed that mice do not develop resistance to nymphs of this tick species and that proliferation of mouse lymphocytes, as well as horse lymphocytes, is inhibited by tick saliva, tick nymphal extract, or tick infestation [17]. Importantly, mouse lymphocytes under the effect of the same *A. cajennense* tick saliva, nymphal extract, or infestation exhibit a predominantly Th-2 cytokine production pattern [17].

Resistance or attempted resistance to ticks is an acquired phenomenon [18-20], and dendritic cells (DCs) – in particular, those of the skin also known as Langerhans cells – play a crucial role in this process [21,22]. Studies published over 30 years ago suggested that Langerhans cells migrate from the skin to the lymph nodes after infestation with ticks of the species *Dermacentor andersoni* and that the presence of these cells in lymph nodes is key for developing tick-specific immunity [21,23-25]. More recently, it has been shown in vivo that ticks of the species *Ixodes scapularis* suppress the immune response in mice, and this effect is dependent on the presence of Langerhans cells [22].

In an attempt to understand how ticks modulate these cells and consequently innate and acquired immune responses, several *in vivo* and *in vitro* studies have shown that tick saliva negatively affects the biology of these cells from their differentiation, migration and maturation until they are able to present antigen and subsequently activate T lymphocytes [26,27]. Despite these findings, most of the studies used the saliva of tick species with very specific host preferences. Regarding the role of DCs during infestation by *A. cajennense* ticks, the only published study demonstrated that after primary and tertiary infestation, significantly fewer CD11c^+^ cells infiltrate the lymph nodes that drain infested skin than CD11b^+^ and CD21^+^ cells do [28], but no information regarding the effects of this saliva on the biology of DCs has been described. Thus, this study evaluated the effects of *A.cajennense* saliva on differentiation, migration and maturation of murine dendritic cells, to understand how the *A.cajennense* saliva of modulates these cells and to identify possible molecules responsible for these effects.

## Methods

### Animals

C57BL/6 (6-8 weeks old) mice were bred and maintained in accordance with protocols established by the ethics committee on animal use in experimental animal facilities of the Federal University of Triângulo Mineiro - UFTM, Uberaba-MG, Brazil. All animal experiments were performed according to protocol 256, a protocol approved by the Ethics Committee on Animal Use (CEUA) of the Federal University of Triângulo Mineiro.

### Reagents

Ultrapure lipopolysaccharide (LPS) from *Escherichia coli* 0111: B4 was obtained from Invivogen (San Diego, CA, USA). Recombinant murine GM-CSF was obtained from Peprotech (Rocky Hill, NJ, USA). The doses of both molecules were determined based on the recommendations of the manufacturer and/or through our concentration-response studies. Cytokines kits (OptEIA™ ELISA), and antibodies were purchased from eBioscience (San Diego, CA, USA) or BD Biosciences (San Jose, CA, USA). MIP-3β and RANTES were obtained from RD System (Minneapolis, MN,USA).

### Saliva collection

Saliva collection was performed as described by Oliveira *et al*. [29], with some modifications. Engorged females were cleaned with phosphate solution in 0.1 M bicarbonate buffer, placed in a dorsal position on tape and then injected with 10-20 μl of 0.2% dopamine solution in PBS, using a 12.7 × 0.33 mm needle (BD Biosciences). Saliva was collected using an automatic pipette and kept on ice. At the end of the collection, saliva was stored at -70°C until it was used in the experiments. The protein concentration was determined on a pool of saliva by the Bradford method (Pierce, Rockford, IL, USA) and the value obtained was ~366 μg/ml.

### Generation of bone marrow-derived DCs

DCs were generated as described previously by Oliveira *et al.* [29], with some modifications. Briefly, bone marrow obtained from femurs and tibiae removed from C57BL/6 mice were cultured in 10 ml RPMI-1640 (GE Healthcare, Uppsala, Sweden) supplemented with 10% v/v inactivated fetal bovine serum (Gibco, Grand Island, NY, USA), 50 mM 2-mercaptoethanol (Sigma), 1 mM sodium pyruvate (Sigma), 25 mM sodium bicarbonate (Gibco), 10 mM HEPES (Sigma), 100 UI/ml penicillin (Sigma), 100 μg/ml streptomycin (Sigma), 25 mM L-glutamine (Gibco), and murine GM-CSF (25 ng/ml). Cells suspensions were prepared at 2.0 × 10^5^ cells/ml. On the fourth day of culture, 10 ml of culture medium supplemented with GM-CSF (50 ng/ml) was added to the plate. After seven days of culture, cells were harvested and their phenotype determined according to expression of CD11b and CD11c by flow cytometry; experiments were continued only when a DC phenotype was confirmed.

### Evaluation of the effect of saliva on the maturation of DCs

Maturation of DCs was assessed by measuring cytokine production (as described below) and by evaluating cell surface expression of stimulatory and co-stimulatory molecules. DCs were distributed into wells at 2 × 10^5^ cells/well, in a 96-well cell culture plate in a volume of 200 μl complete RPMI. Cells were then incubated for 1 hour with different concentrations of saliva (1:10, 1:30, 1:100, 1:300, 1:1000 v/v), after which they were stimulated with LPS (100 ng/ml) for 18 hours. The cells were collected and analyzed by flow cytometry for expression of MHC-II, CD40, CD80, CD86 and PD-L1.

### Evaluation of the effect of saliva on the expression of chemokine receptors

DCs were differentiated as described above and distributed into wells at 2 × 10^5^ cells/well. They were then pre incubated for 1 hour with different concentrations of saliva (1:30 and 1:100 v/v) and divided into two groups: those that were stimulated with LPS (100 ng/ml) for 18 hours and those that were not stimulated with LPS. The expression of CCR7 and CCR5 chemokine receptors on the cell surface were then assessed by flow cytometry.

### Evaluation of the effect of saliva on the differentiation of DCs

Cells were collected from the bone marrow and distributed at 2 × 10^5^ cells/well in a 48-well plate in an initial volume of 200 μl of complete RPMI plus GM-CSF (25 ng/ml). Different concentrations of saliva (1:30, 1:100, 1:300, 1:1000 v/v) were added to the wells and again on the third day of culture. On the fourth day, 200 μl complete RPMI with GM-CSF (50 ng/ml) was added. On the fourth and seventh days of culture, cells were assessed for expression of CD11c, CD11b, CD40, CD80, CD86 and MHC-II by flow cytometry.

### Evaluation of the effect of saliva on the expression of CD11b and CD11c in differentiated DCs

The effect of saliva was also evaluated on cells that had already differentiated, to determine if tick saliva would be able to change the phenotype of these cells. DCs were distributed at 2 × 10^5^ cells/well in a 96-well plate, in a volume of 200 μl of complete RPMI. They were incubated for 18 hours with different concentrations of saliva (1:10, 1:30, 1:100, 1:300, 1:1000 v/v). The cells were then collected and assessed for expression of CD11b and CD11c by flow cytometry.

### Flow cytometry analysis

The cultured cells were analyzed by flow cytometry using antibodies for CD11c, CD11b, CD40, CD80, CD86, MHC-II, PD-L1, CCR5 and CCR7 conjugated to phycoerythrin (PE), phycoerythrin-cyanine (PE-Cy7), fluoresceinisothiacyanate (FITC) or allophycocyanine (APC). Data were acquired using a FACSCalibur (BD15 Immunocytometry Systems) with CellQuest 5.1 software (BD Biosciences) and analyzed with FlowJo software (Tree Star Inc., Ashland, OR, USA). The results were expressed in relative percentage given the frequency or the medium fluorescence intensity (MFI).

### Cytokine assays

The cytokines IL-10, IL-6, IL-12p40 and TNF-α were evaluated by enzyme-linked immunosorbent assay (ELISA) (“sandwich” type) using pairs of monoclonal antibodies, according to the manufacturer’s instructions (BD Bioscience). For the samples of IL-6 and IL-12p40, dilutions of 10 and 20 times were carried out, respectively.

Concentrations of cytokines were determined by interpolating their absorbance values into a standard curve prepared with known concentrations of murine recombinant cytokines -using the StartView program- and expressed as pg/ml.

### Apoptosis

DCs were distributed at 2 × 10^5^ cells/well in a culture plate of 96 wells, in a volume of 100 μl of complete RPMI. Cells were incubated for 18 hours with different concentrations of saliva (1:10, 1:30, 1:100, 1:300, 1:1000 v/v), then collected, washed, and resuspended in Annexin buffer. The assay was performed using Annexin V-fluorescein isothiocyanate (Annexin V-FITC; 2.5 μg/ml) and propidium iodide (PI; 2.5 μg/ml), according to manufacturer’s specifications (BD Pharmigem). Annexin V^−^ PI^−^ cells were considered viable cells. The data were obtained with a FACSCalibur flow cytometer with CellQuest 5.1 software and were analyzed using FlowJo software. DCs cultured only with culture medium were used as a positive control of cell viability, and DCs maintained at 57°C for 30 minutes were used as a positive control of cell death.

### Evaluation of the effect of saliva on cell migration in the Boyden chamber

Tests were performed using a Boyden chemotaxis chamber (NeuroProbe, Cabin John, MD, USA) with a polycarbonate membrane having a pore size of 5 micrometers. The migration stimulants MIP-3β (CCL19) and RANTES (CCL5) (in concentrations of 500 ng/ml and 50 ng/ml, respectively) (R&D Systems) were placed into the lower wells of the plate. A suspension of cells at a concentration of 1×10^6^ cells/ml was placed in the wells, with each well containing cells that were either untreated or pretreated with saliva for 18 hours at different concentrations (1:30 and 1:100 v/v) in the presence or absence of LPS (100 ng/ml) were placed into the upper part. The chemokine RANTES is specific for receptors present especially on immature DCs, including CCR5. The chemokine MIP-3β is specific for the CCR7 receptor and is present mainly in mature DCs. After a 1.5 hour incubation at 37°C in a humidified 5% CO2 incubator, the plate was disassembled and the membrane was removed, fixed and stained with Diff-Quik (Baxter Diagnostics, Düdingen, Switzerland). The analysis was performed by an optical microscopy lens with 100× magnification. Five different fields were counted per triplicate, making a total of 15 fields per treatment. The results are given in average ± SD number of migrated cells.

### Prostaglandin concentration determination

To determine the concentration PGE_2_ in each saliva sample, EIA kit was used according to the manufacturer’s instructions (Enzo Life Sciences, NY, USA). The analysis was performed at an absorbance at 405 nm with correction between 570 and 590 nm in a spectrophotometer. The concentration of prostaglandin was determined by comparison with a standard curve prepared according to the kit manufacturer’s instructions. The detection limit for this assay was 13.5 pg/ml.

### Statistical analysis

Statistical analysis was performed using GraphPadPrism 5.0 (GraphPad Software, San Diego, CA, USA). For data with a Gaussian distribution, ANOVA and a Tukey post-test were performed; for data with a non-Gaussian distribution, a Kruskal-Wallis test with a Dunns post-test were performed. Bar graphs were used to show the mean and standard deviation of each numerical result. The results were considered significant when the p value was 0.05 (5%) or less.

## Results

### Saliva inhibits dendritic cell differentiation

**Figure 1:**
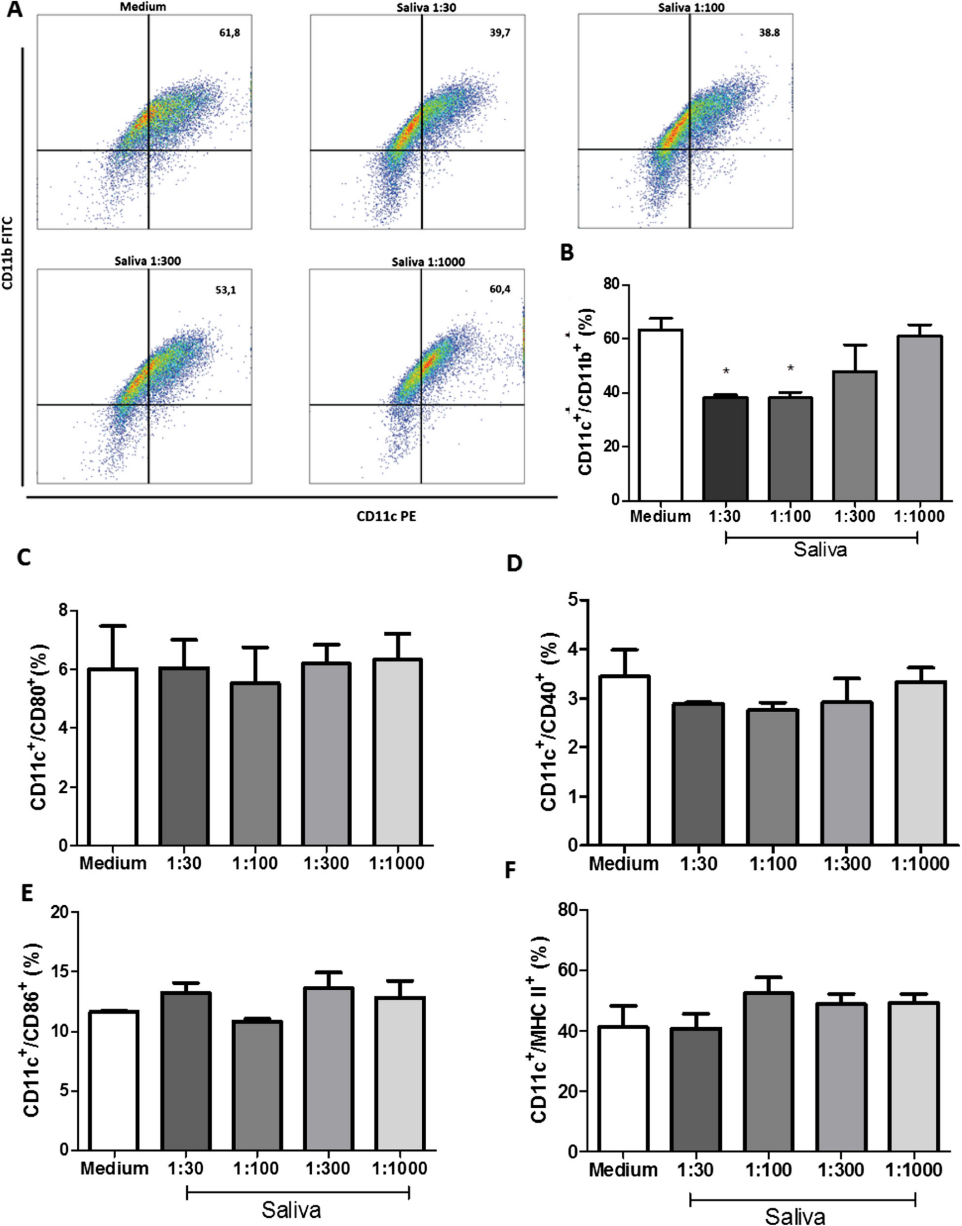
**Effect of tick saliva on the differentiation of DCs.** Bone marrow cells derived from C57BL/6 mice were cultured with GM-CSF (25 ng/ml) in the presence or absence of tick saliva for 7 days. Plots of DCs on the 7^th^ day, as evaluated by CD11c and CD11b evaluation expression **(A)**. Percentage of DCs on the 7^th^ day, as evaluated by CD11c and CD11b expression **(B)**. Cultured cells were collected and evaluated for the expression of surface molecules CD11c/ CD80 **(C)**, CD11c/CD40 **(D)**, CD11c/CD86 **(E)** and CD11c/ MHC-II **(F)** by flow cytometry. Bars represent the mean ± SD percentage of DCs expressing molecular markers from triplicate experiments. * *p* <0.05 compared with cells cultured without saliva (labeled “Medium” in the graph).

The effect of saliva on the differentiation of DCs was analyzed from the culture of bone marrow precursor cells of mice in the presence or absence of different concentrations of saliva (1:30, 1:100, 1:300 and 1:1000 v/v). The percentage of differentiation was evaluated both on the fourth day and on the seventh day. Our data show that, despite inducing a slight decrease in differentiation, saliva was not able to significantly reduce this process on the fourth day of evaluation at any concentration tested (data not shown). However, on the seventh day, saliva inhibited the differentiation of precursor bone marrow cells into DCs and CD11c^+^ CD11b^+^ cells, leading to a suppression of 35,8% and 37,2% (*p* <0.05) at dilutions of 1:30 and 1:100, respectively, when compared with the cells differentiated in the absence of saliva (Figure 1A-B). When evaluating the expression of CD80, CD40, CD86 and MHC-II in cells cultured for seven days, with or without saliva, results demonstrated that saliva does not induce significant changes on CD11c^+^ differentiated DCs (Figure 1C-F).

### Effect of tick saliva on DCs that have already differentiated into CD11c^+^ CD11b^+^ cells

**Figure 2:**
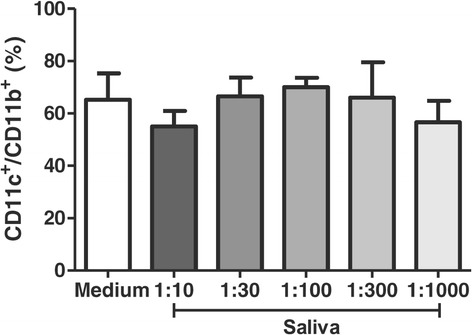
**Effect of tick saliva on differentiated DCs.** Bone marrow cells derived from C57BL/6 mice were cultured with GM-CSF (25 ng/ml) for 7 days. Immature DCs were then obtained and incubated with different dilutions of saliva (1:10, 1:30, 1:100, 1:300 and 1:1000 v/v) for18 hours. The expression of CD11c and CD11b were evaluated. Bars represent the mean ± SD percentage of DCs expressing molecular markers from duplicate experiments. * *p* <0.05 compared with cells cultured without saliva (labeled “Medium” in the graph).

After observing that saliva interferes with differentiation of bone marrow cells into DCs, we also assessed the ability of saliva to induce cellular plasticity, the ability of cells to change their phenotype. Cells that had already differentiated into DCs were incubated for 18 hours with saliva in different concentrations (1:10, 1:30, 1:100, 1:300 and 1:1000 v/v) and the expression of CD11b and CD11c was measured. *A.cajennense* saliva did not alter the expression of CD11b and CD11c in cells that had already differentiated. On average, 65% of DCs that were not treated were double positive for CD11b and CD11c; there were no significant differences (*p* >0.05) between the percentages of double positive cells among those cells treated with different concentrations of saliva (Figure 2). Likewise, the treated and untreated DCs showed no significant differences with respect to the percentages of different populations of CD11c^+^/CD11b^−^ and CD11c^−^/CD11b^+^ cells (data not shown).

### Saliva inhibits expression of surface molecules on DCs stimulated with LPS

**Figure 3:**
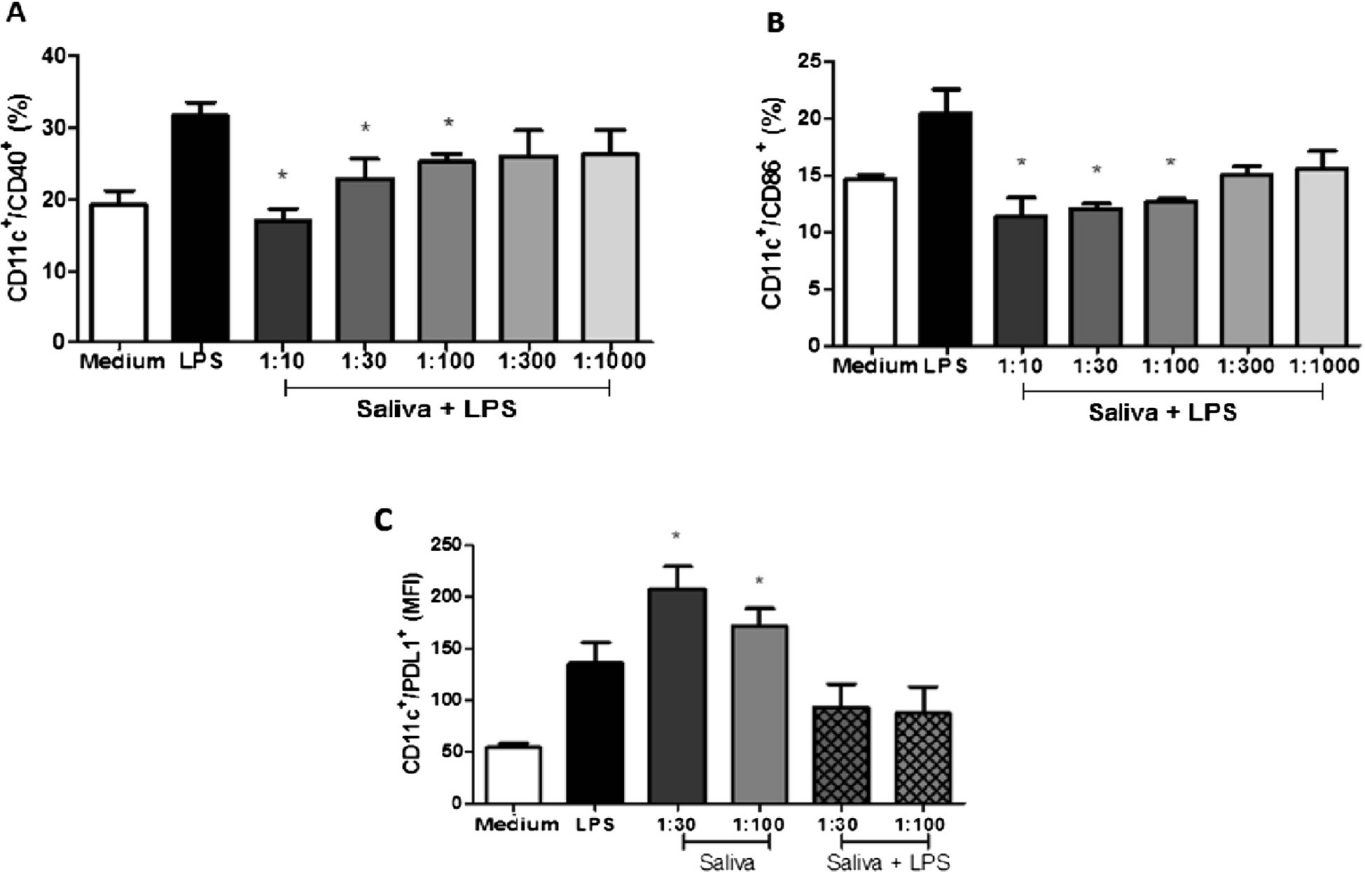
**Effect of tick saliva on the expression of CD40, CD86 and PD-L1 stimulated with LPS.** Bone marrow cells derived from C57BL/6 mice were collected and cultured for 7 days in the presence of GM-CSF to allow differentiation into DCs. DCs were then pre-incubated with saliva (diluted 1:10, 1:30, 1:100, 1:300 and 1:1000 v/v) from *A. cajennense* for 1 hour. After that, DCs were then stimulated for an additional 18 hr with LPS (100 ng/ml). After this time, culture cells were collected and evaluated for the expression of surface molecules CD11c/ CD40 **(A)**, CD11c/CD86 **(B)**, and CD11c/PD-L1 **(C)**. Bars represent the mean ± SD percentage of DCs expressing molecular markers from triplicate experiments. *: *p* < 0.05 compared with DCs cultured with LPS.

The expression of stimulatory and co-stimulatory molecules CD40, CD86, CD80 and MHC-II was evaluated in dendritic cells incubated with or without saliva for 1 hour and subsequently stimulated with LPS (100 ng/ml) for 18 hours. A 1:10 dilution of saliva inhibited the expression of CD40 by 46.7% compared to cells that were not incubated in saliva. 1:30 and 1:100 dilutions of saliva also showed significant results, with inhibitions of 27.8% and 20.5% on average, respectively. The inhibition of CD86 expression in DCs was 44.3%, 41% and 38% for dilutions of 1:10, 1:30 and 1:100, respectively. All the results above showed significant differences (*p* <0.05) compared to cells treated only with LPS. Cells incubated with1:300 and 1:1000 dilutions of saliva did not inhibit expression of any of these molecules (Figure 3A-B). Expression of CD80 and MHC-II was not significantly altered in the presence of saliva at any of the tested concentrations (data not shown). PD-L1 is a molecule expressed by DCs that has an inhibitory profile. Its expression is increased in DCs with tolerogenic features. Flow cytometry was performed to assess the expression of PD-L1 on DCs cultured with saliva, with or without LPS stimulation. As shown in Figure 3C, cells incubated only with saliva showed a significant increase in the expression of PD-L1, while those incubated with saliva and stimulated with LPS showed no change at significant levels. PD-L1 expression experiments were performed only for 1:30 and 1:100 dilutions of saliva because of limited amounts of biological sample.

### Saliva inhibits production of pro-inflammatory cytokines and stimulates IL-10 production

**Figure 4:**
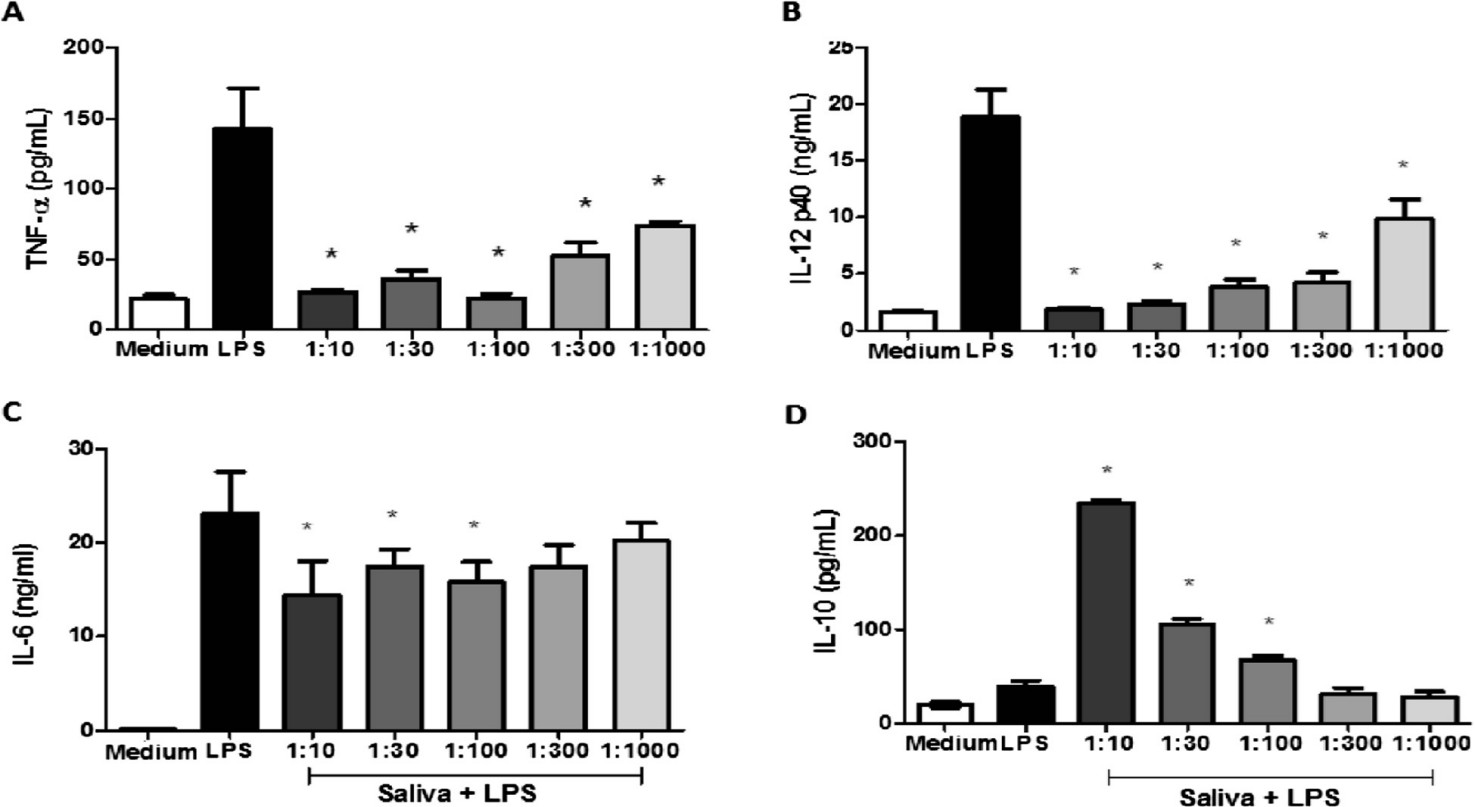
**The effect of tick saliva on TNF-α, IL-12 p40, IL-6 and IL-10 production in DCs stimulated with LPS.** Bone marrow cells derived from C57BL/6 mice were collected and cultured for 7 days in the presence of GM-CSF to allow differentiation into DCs. DCs were then pre-incubated with saliva (diluted 1:10, 1:30, 1:100 1:300 and 1:1000 v/v) from *A. cajennense* for 1 hour. DCs were then stimulated for an additional 18 hours with LPS (100 ng/ml). The culture supernatant was then collected and analyzed to detect TNF-α **(A)**, IL-12 p40**(B)**, IL-6 **(C)** and IL-10 **(D)** by ELISA. Bars represent the mean ± SD production level of TNF-α, IL-12 p40, IL-6 and IL-10 in cultured DCs from triplicate experiments. *: *p* < 0.05 compared with DCs cultured with LPS.

To assess if saliva interferes with the production of cytokines, DCs were exposed to different concentrations of saliva and maturation was subsequently stimulated by incubating with LPS (100 ng/ml) for 18 hours. As shown in Figure 4, TNF-α (A), IL-12p40 (B) and IL-6 (C) production were significantly inhibited (*p* <0.05) in the presence of saliva compared to cells treated with only LPS. The production of both cytokines TNF-α and IL-12p40 was inhibited up to 81% and 90%, respectively, when incubated with increasing concentrations of saliva, from 1:1000 to 1:10 dilution.

The highest dilution capable of inhibiting IL-6 production was 1:100, which was associated with a 34% inhibition. IL-10 production was significantly (*p* <0.05) enhanced by the presence of saliva (Figure 4D). Stimulation of IL-10 production was dose-dependent: an up to 6-fold increase was observed with a 1:10 dilution of saliva and a1.5-fold increase was observed with a 1:100 dilution. Cells cultured in the presence of saliva only showed no significant changes in the production of any of the measured cytokines (data not shown).

### Effect of A. cajennense saliva on migration and expression of CCR5 and CCR7 by DCs

**Figure 5:**
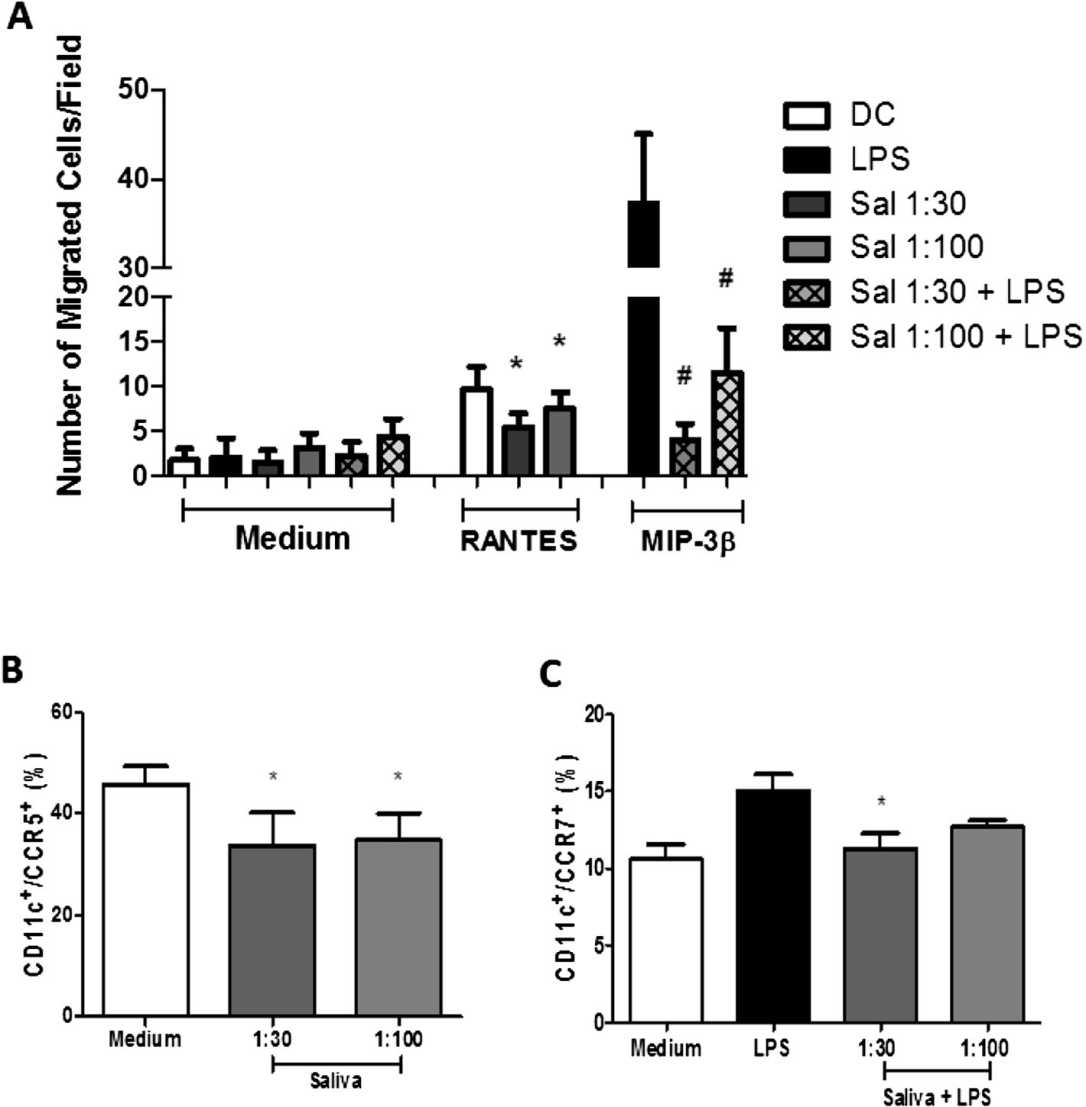
**The effect of tick saliva on migration and expression of CCR5 and CCR7 in DCs.** Bone marrow cells derived from C57BL/6 mice were collected and cultured for 7 days in the presence of GM-CSF to allow differentiation into DCs. DCs were then pre-incubated with saliva (diluted 1:30, 1:100 v/v) from *A. cajennense* for 1 hr. DCs were then stimulated for an additional 18 hours with LPS (100 ng/ml). A Boyden microchamber migration assay was then performed with 2x 10^6^ cells/ml seeded into the upper wells and the buffer control (medium) or chemokines (RANTES and MIP-3β) seeded into the lower wells of the microchamber. The incubation period was 1.5 h in a humidified 5% CO2 incubator at 37°C. The filters were then removed and stained and the number of migrated cells was counted **(A)**. Cultured cells were collected and evaluated for the expression of surface molecules CD11c/ CCR5 **(B)**, CD11c/CCR7 **(C)**. In **(A)**, bars represent the mean ± SD number of migrated cells in 15 high power fields from duplicate experiments; in **(B)** and **(C)**, bars represent the mean ± SD percentage of DCs expressing molecular markers from duplicate experiments. *: *p* <0.05 compared with DCs cultured with LPS.

To determine whether saliva can alter the migration of DCs, we used a Boyden chamber and evaluated whether incubation with saliva altered DC expression of receptors for RANTES and MIP-3β. The Boyden chamber comprised two parts: the lower part, to which RANTES and MIP-3β were added, and the upper part, to which DCs treated with saliva were added. Untreated DCs were used as controls for immature cells, which migrate toward RANTES, and DCs treated with LPS were used as a control for activated cells, which migrate toward MIP-3β. Our results showed a significant reduction in the migration of immature DCs and activated DCs when they were treated with saliva. Inhibition of migration toward RANTES was 43% and 22% for immature cells incubated with 1:30 and 1:100 dilutions, respectively, of saliva. Migration toward the chemokine MIP-3β was inhibited by 88% and 69% (for 1:30 and 1:100 dilutions, respectively, of saliva) for cells activated with LPS (Figure 5A). The observed inhibition of migration of immature cells toward RANTES and of activated cells toward MIP-3β prompted us to assess if these results were due to altered expression of receptors for these chemokines. Thus, the expression of the receptors CCR5 and CCR7 was analyzed by flow cytometry. Our results showed that the expression of both receptors was significantly reduced. In immature cells cultured with saliva, CCR5 expression was reduced by approximately 26% and 24% for 1:30 and 1:100 dilutions, respectively (Figure 5B). In cells cultured with saliva and activated with LPS, CCR7 expression was reduced by 25% and 15% for 1:30 and 1:100 dilutions, respectively; no significant reductions were observed with lower dilutions (Figure 5C).

### *Saliva of* A. cajennense *does not reduce DC viability* in vitro

**Figure 6:**
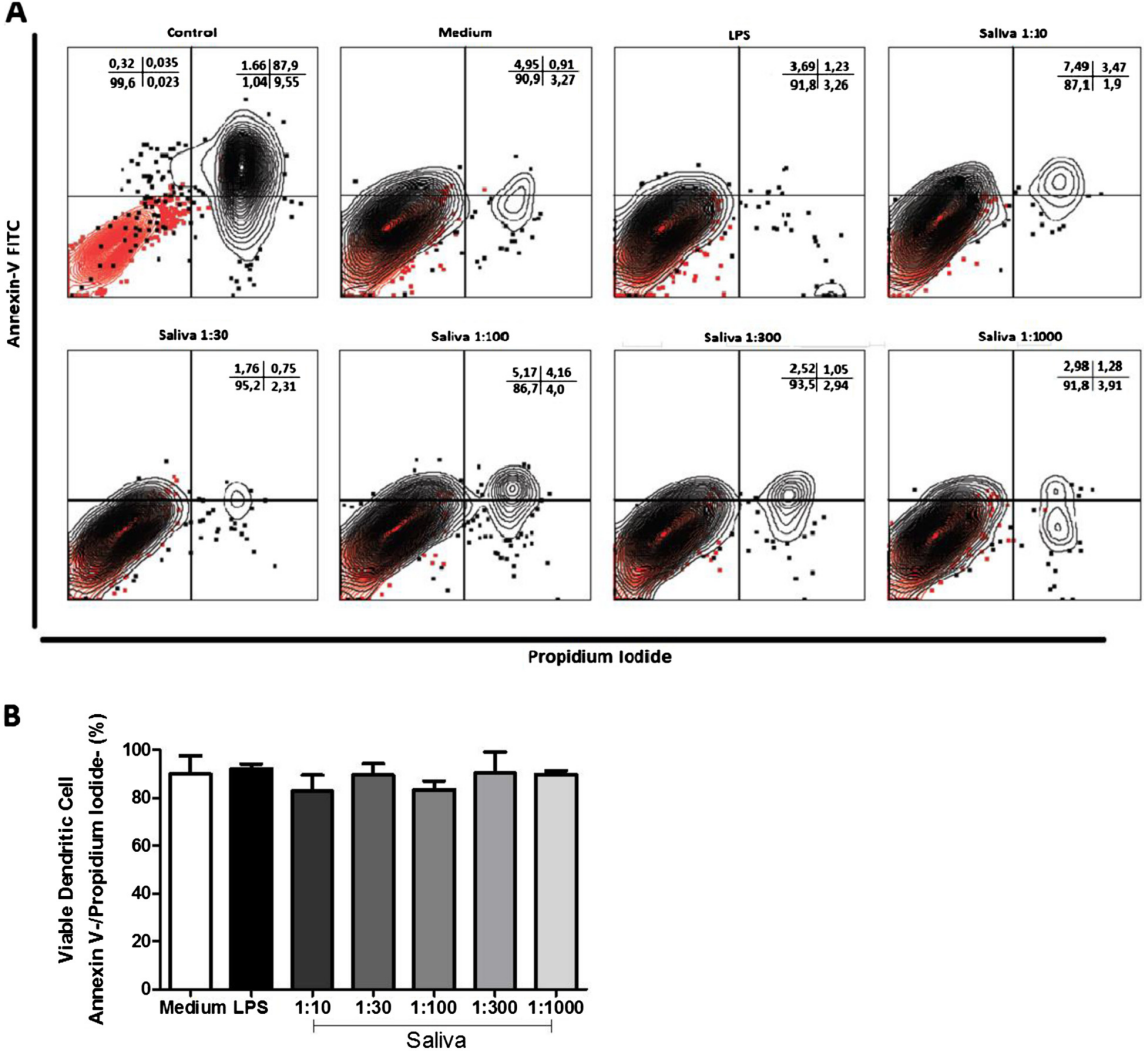
**The effect of tick saliva on viability of DCs.** Bone marrow cells derived from C57BL/6 mice were collected and cultured for 7 days in the presence of GM-CSF to allow differentiation into DCs. DCs were then incubated with either saliva (diluted 1:10, 1:30, 1:100 1:300 and 1:1000 v/v) from *A. cajennense* for 18 hours. After that, the cells were collected and stained with Annexin V and propidium iodide to evaluate apoptosis by flow cytometry. Cell viability is shown in dot plots **(A)**, where negative control cells are colored red and Annexin-V-FITC and Propidium Iodide stained cells are colored black **(B)**. Bars represent the mean ± SD number of viable DCs (Annexin V-/PI-) from duplicate experiments.

The effects of saliva on DCs – reduced differentiation, expression of surface molecules and cytokine production – could be due to induction of cell death. To evaluate this possibility, DCs that had been cultured for 7 days were incubated with different concentrations of saliva (1:10, 1:30, 1:100, 1:300, 1:1000 v/v) for 18 hours and stained with Annexin V and Propidium Iodide. DCs that were negative for both stains were considered viable cells. As a positive control for cell death, DCs that were not incubated with saliva were maintained at 57°C for 30 minutes, as represented in Figure 6A. Our results shows that saliva does not significantly reduce the viability of DCs *in vitro* in any of the concentrations tested (Figure 6B).

### A. cajennense *saliva contains prostaglandin (PGE*_*2*_*)*

According to data reported in the literature, saliva from some tick species including *Rhipicephalus sanguineus, Dermacentor variabilis, Ixodes dammini,* and *Ixodes scapularis* may contain PGE_2_ [29-32]. We therefore performed PGE_2_ quantification by competitive enzyme-linked immunosorbent assay and found a concentration of approximately 80 ng/ml PGE_2_ in *A. cajennense* saliva (mean 80,2 ng/ml from triplicate experiments).

## Discussion

The tick *A. cajennense* parasitizes more than one host to complete its life cycle, has low host specificity and is a species with aggressive hematophagism habits due to the size of its mouthparts. Despite these characteristics, it has an extraordinary ability to evade host defense mechanisms, which favors their infestation and subsequent transmission of pathogens. In this paper, we provide the first report that *A. cajennense* saliva modulates the biology of DCs, a major type of immune skin cell. We have shown that the saliva of *A.cajennense* interferes with DC differentiation, migration, expression of stimulatory and co-stimulatory molecules and cytokine production. These effects are not related to toxic activity of the saliva because *A. cajennense* saliva does not reduce viability of DCs in any of the dilutions tested.

First, it was demonstrated that *A. cajennense* saliva inhibits differentiation of DCs, although the expression of stimulatory and co-stimulatory molecules on DCs remained unchanged. These results indicating inhibition of differentiation are consistent with findings previously described for other tick species. The saliva of the tick *R. sanguineus* inhibits differentiation of DCs and this effect is dependent on PGE_2_ in the saliva [29,33]. The saliva of the tick *R. appendiculatus* inhibits differentiation of human DCs from monocytes; however, the molecule responsible for this effect is a protein named Japanin [34]. In contrast to the saliva of ticks studied so far, the saliva of *Aedes aegypti* does not inhibit the differentiation of DCs [35]. These results from the literature demonstrate that such modulation of DCs does not occur with all bloodsucking arthropods, but seems to occur with ticks because many tick species have been reported to contain molecules with DC-modulating activity in their saliva [36,37]. Our finding that the expression of co-stimulatory and stimulatory molecules was unchanged in those DCs that were able to differentiate differs from the findings of Cavassani *et al.* [33], who found a reduced expression of CD80 and CD86 on DCs that differentiated in the presence of saliva of *R. sanguineus*. It has also been reported that saliva of the tick *R. sanguineus* inhibits the expression of both CD11b and CD11c on DCs that have differentiated [33]. In our work, no decrease in the population of already differentiated DCs or change in the expression of CD11b or CD11c was observed. Taken together, these data suggest that saliva-induced inhibition of differentiation may contribute to the successful feeding of ticks because such an inhibition leads to a decrease in the number of antigen-presenting cells in the bite site and thereby reduces the activation of the acquired immune response, providing a favorable environment for ectoparasites [29]. A study by Vesely *et al.* [22] showed that mice with a deficiency in Langerhans cell (skin DCs) have difficulty in suppressing the Th1 response and therefore have an increased susceptibility to tick infestation.

It is widely known that maturation of DCs can be assessed by the increased expression of surface molecules such as CD40, CD80, CD86, MHC-II and PD-L1, as well as the increased production of pro-inflammatory and anti-inflammatory cytokines. With regard to the expression of co-stimulatory or co-inhibitory molecules, it was found that the saliva of *A.cajennense* reduces the expression of CD40 and CD86 while stimulating the expression of PD-L1 on DCs. The expression of MHC-II and CD80 remained unchanged. The observed inhibition of the expression of CD40 and CD86 are consistent with previously published results [29,33,38] where DCs stimulated with LPS showed reduced expression of CD40, CD80 and CD86 in the presence of saliva from *R. sanguineus*. Furthermore, the saliva of *R. appendiculatus* contains Japanin, which inhibits CD86 and CD83, thereby decreasing the maturation process [34]; saliva of *Ixodes scapularis* inhibits CD40, CD80 and CD86, and this effect is attributed to activity of Sialostatin L [31,39]; *R. microplus* saliva also alters the expression of CD80, CD86 and CD69 on macrophages [40].

PD-L1 (programmed death ligand 1 or B7-H1, CD274) is a member of the family of inhibitory molecules that are present in DCs and bind to PD-1 (Programmed Death 1), which is transiently increased by T lymphocytes during antigen presentation. This connection, when it occurs, leads to disruption of TCR signaling [41,42]. The regulation of this signaling can lead to a state of exhaustion of T cells, which is favorable to pathogens because of the corresponding deficiency in immune response, including decreased production of cytokines [43,44]. Our results show an increased expression of PD-L1 in DCs stimulated with saliva. Thus we suggest that *A. cajennense* saliva can interfere with antigen presentation by both inhibiting co-stimulatory and stimulatory molecules as well as inducing inhibitory molecules such as PD-L1, which results in lower T cell activation. CD11c^+^ DCs that express CD80 and CD86 at low levels and PD-L1 at high levels may have impaired ability to present antigen. Impairment of antigen presentation is believed to be one of the most important mechanisms of modulation by tick saliva. As an example, lack of CD40 and CD86 induces an anti-inflammatory response, characterized by induction of apoptosis or anergy of T cells [38,45,46].

*A.cajennense* saliva also had many remarkable effects on the production of cytokines. Production of pro-inflammatory cytokine IL-12p40, IL-6 and TNF-α was reduced in DCs cultured with saliva and stimulated with LPS, while production of the anti-inflammatory cytokine IL-10 was significantly increased. Similar data were observed in several other studies on tick saliva [29,31,33,34,38,39,45]. IL-6 is considered an important pro-inflammatory cytokine; it is actively produced during acute phases of the inflammatory response and drives the inflammation during the transition from the innate to the adaptive immune responses [47,48]. IL-12 and TNF-α are responsible for promoting the cells toward a Th1 profile, which is unfavorable to the parasite. Our findings show a decrease in the production of these cytokines, which contributes to the development of a Th2 profile, which favors the tick remaining in the host [45,49,50]. The increase of IL-10 production by DCs, in addition to generating an anti-inflammatory environment, can direct the activation of regulatory T lymphocytes [51]; these effects of increased IL-10 production may regulate the activation of naive lymphocytes to inhibit the expression of CD80/86 by DCs [46,52].

Migration of immature DCs to peripheral tissues or mature DCs to secondary lymphoid organs are key events in the induction of the innate and acquired immune response, respectively. Immature DCs migrate by chemotactic activity induced by chemokines such as RANTES, while mature DCs migrate by chemotactic activity induced by MIP-3β [53-57]. In our studies, we observed that DCs cultured with saliva exhibited reduced migration toward both RANTES as MIP3β. Our finding regarding RANTES agrees with previously published results on *R. sanguineus* saliva [58], but inhibition of migration toward the MIP-3β result had not previously been shown for tick saliva.

The migration of DCs toward RANTES and MIP-3β depends on the expression of CCR5 and CCR7 receptors, respectively. We evaluated the effect of saliva on the expression of these surface molecules. Our findings show that the inhibition of migration is linked to decreased expression of these receptors because surface expression of both receptors is reduced on DCs cultured with saliva for 18 hours. This event seems to be an escape mechanism often used by endo-and ectoparasites to circumvent the responses of their hosts [57]. *Leishmania major* can inhibit the expression of the chemokine receptors CCR2 and CCR5 [59], as can cytomegalovirus, which internalizes CCR5 and CCR1 [60]. Studies have demonstrated that tick saliva can induce down-regulation of CCR5 both *in vitro* and *in vivo* [45,58]. These findings may help explain the reduction of DCs at the site of the bite. We found a decrease in the expression of the CCR7 receptor in LPS-stimulated mature DCs exposed to saliva, which explains the inhibition of migration in response to MIP-3β. These results for CCR7 never were descripted in papers with saliva of ticks. CCR7 is expressed on activated DCs, which migrate toward the lymph nodes to activate lymphocytes and initiate the adaptive immune response. Some pathogens are capable of evade the host immune defense by interfering the host expression of the CCR7. Cytomegalovirus induces low expression of CCR7 and reduces migration of DCs to lymph node, impairing activation of the adaptive response mechanism; these effects have also been observed in infections with the Herpes Simplex (HSV-1), human respiratory syncytial virus (HRSV), human metapneumovírus (HMPV) and measles virus [56,61-63]. Some pro-inflammatory cytokines produced during maturation are related to the expression of CCR7 by DCs. Our results indicated a reduction in the production of TNF-α, as previously mentioned. The low production of TNF-α or reduction in the expression of its receptors may be related to decreased expression of CCR7 [64,65]. Thus, the regulation of migration of activated DCs to the lymph node is associated with the ability to delay the activation of an efficient adaptive response, which is extremely advantageous to a feeding tick.

We believe that some of the results reported here might be attributed to the presence of PGE_2_ in *A cajennense* saliva. As mentioned, PGE_2_ has been identified in the saliva of a number of bloodsucking arthropods [26,37]. Prostaglandins are the most abundant molecules identified in tick saliva to date [36], and they are important lipid mediators of immune responses. The relationships between PGE_2_ and DCs have been extensively studied. DC differentiation can be altered in the presence of PGE_2_ through EP2/EP4 receptors, and PGE_2_ can influence cytokine production toward a predominantly anti-inflammatory profile. PGE2 also inhibits the migration of DCs, interferes with the proliferation and activation of T lymphocytes, and induces a Th2 response, which in turn is favorable to ticks [26,28,29,63-66]. On the other hand, other immunosuppressive effects of the *A. cajennense* saliva observed in this work, such as the inhibition of CCR7 expression, subsequent DCs migration and increase in PD-L1 expression cannot be yet attributed to PGE_2_ activity. Future studies are needed to test this hypothesis. In other words, we believe that PGE_2_ is not the only molecule with immunomodulatory properties in *A. cajennense* tick saliva. Our results might be explained by the concentration of PGE_2_ found in the saliva of different species of ticks. For example, PGE_2_ concentrations in *I. scapularis* saliva are much higher (505 ng/ml) than what is found in the saliva in our study (80 ng/ml) [31]. In the case of saliva from *R. sanguineus,* that has low concentration of PGE_2_, this low concentration is compensated by the presence of adenosine that collaborate with saliva to reach immunodulatory activities in pharmacological levels [29]. So, we suggest that the *A. cajennense* tick saliva has more molecules with immunological effects, but other studies must be done to prove this proposal. Thus, the future perspective is identify and isolate other molecules in the saliva, such as adenosine, and evaluate whether these molecules may modulate other immune system components such as cells, cytokines and chemokines so as to facilitate its feeding and spread of pathogens transmitted by them.

## Conclusions

Our studies are the first to report the effect of saliva of *A. cajennense* on the biology of DCs besides identifying significant amounts of PGE_2_. These findings are relevant to the understanding of mechanisms used by these arthropods to modulate tick-host interactions. This study will aid in the search for new alternatives to control these pests and the pathogens they transmit.
